# A cluster of four cases of cutaneous leishmaniasis by *Leishmania donovani* in Cyprus: a case series

**DOI:** 10.1186/1752-1947-8-354

**Published:** 2014-10-24

**Authors:** Maria G Koliou, Yiannakis Antoniou, Maria Antoniou, Vasiliki Christodoulou, Apostolos Mazeris, Elpidoforos S Soteriades

**Affiliations:** 1 Archbishop Makarios Hospital, Department of Paediatrics, 6 Korytsas St., Strovolos, 1474 Nicosia, Cyprus; 2 Cyprus Institute of Biomedical Sciences (CIBS), Nicosia, Cyprus; 3 Private veterinary practitioner, 8820 Polis Chrysochous, Cyprus; 4 Laboratory of Clinical Bacteriology, Parasitology, Zoonoses and Geographical Medicine, Faculty of Medicine, University of Crete, Voutes, Heraklion, 71003 Crete, Greece; 5 Ministry of Agriculture, Veterinary Services, Nicosia, Cyprus; 6 Department of Environmental Health, Environmental and Occupational Medicine and Epidemiology (EOME), Harvard School of Public Health, Boston, MA, USA

**Keywords:** Cluster, Cutaneous leishmaniasis, Cyprus, *Leishmania donovani*

## Abstract

**Introduction:**

Leishmaniasis is endemic in more than 95 countries and is the only tropical/subtropical vector-borne disease that has been endemic in Southern Europe for decades. To the best of our knowledge, this is the first case of cutaneous leishmaniasis by *Leishmania donovani* in a child and the first cluster with adult cases reported in Europe.

**Case presentation:**

We describe a familial cluster of four cutaneous leishmaniasis cases among Greek Cypriots caused by *L. donovani* in a Paphos village, in Cyprus. A 6-year-old boy (Case number 1) had a persistent lesion in the left angle of his upper lip, a 60-year-old woman (Case number 2) presented with a 2cm-diameter glabella lesion on her forehead, a 60-year-old man (Case number 3) developed a lesion on his moustache area and a 40-year-old woman (Case number 4) had a lesion on her neck. In Case number 3 the lesion was self-cured; the other cases recovered after surgical resection followed by liposomal amphotericin B (Case numbers 1 and 4) or thermotherapy and liposomal amphotericin B (Case number 2).

**Conclusions:**

This familial cluster of cutaneous leishmaniasis, due to the anthroponotic *L. donovani,* shows that the sand fly species responsible for transmitting this parasite species is found in the area around the three neighbouring houses involved. The factors favourable for the survival, spread and contact of the vector with people could be assessed in this area for the establishment of preventative measures to safeguard public health.

## Introduction

Leishmaniasis, a vector-borne disease, is caused by protozoans of the genus *Leishmania* and is endemic in 95 countries [1-4]. Transmission may be zoonotic or anthroponotic, by the bite of female phlebotomine sand flies. It includes a wide spectrum of manifestations ranging from localised ulcerative lesions at the site of the sand fly bite (localised cutaneous leishmaniasis, CL) to the potentially fatal disseminated visceral form (visceral leishmaniasis, VL).

In Southern Europe, until recently, two species were identified as causing leishmaniasis in humans. One of these species is *Leishmania infantum,* the most widely circulating species, causing over 90% of the cases of zoonotic CL and VL. The other is *Leishmania tropica* causing anthroponotic CL, encountered sporadically in Greece and in eastern Mediterranean countries. In Cyprus, only two human cases of VL were reported between 1935 and 2006 [5]. However, in 2006 six cases of CL and VL were detected in humans and since then sporadic cases of CL and VL are reported annually [6]. All cases have been associated with the anthroponotic *Leishmania donovani* species.

We present the first familial cluster of CL cases due to *L. donovani,* in Cyprus.

## Case presentation

**Figure 1:**
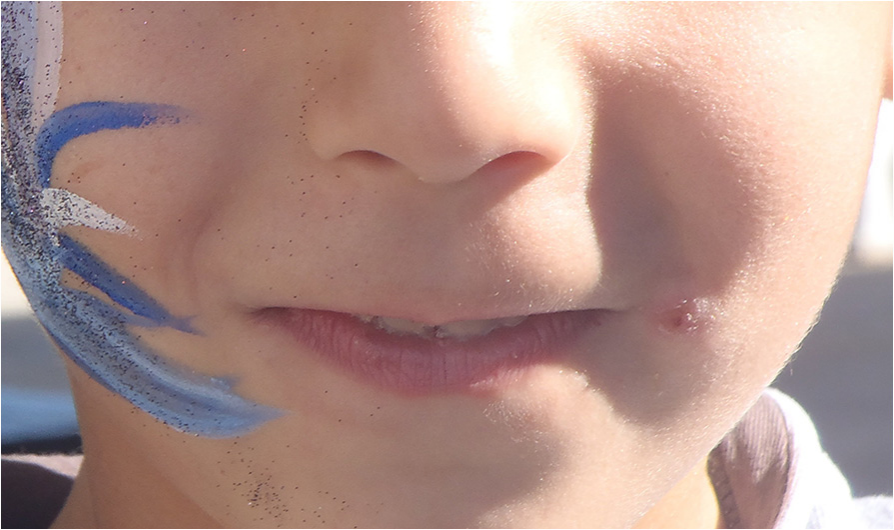
The child’s lesion before treatment.

A child and three adults from an extended family, who lived in neighbouring houses, were involved in a cluster of CL detected in a small village in the district of Paphos, where most cases of leishmaniasis in Cyprus since 2005 have been diagnosed.Case number 1, a 6-year-old Greek Cypriot boy, was referred to our hospital because of a small ulcerative lesion next to the left angle of his lips (Figure 1). The lesion persisted for a period of 11 months prior to referral despite several courses of oral antibiotics or local antibiotic ointments. The child never complained of fever, chills, sweating or weight loss during this time. His previous history was unremarkable.

On examination he had a shallow ulcer right next to the left angle of his lips with a diameter of approximately 1cm. There were no enlarged lymph nodes and no hepatosplenomegaly. A more detailed examination of his ear, nose and throat did not reveal any mucosal involvement of the lesion. His full blood count and inflammatory markers (erythrocyte sedimentation rate and C-reactive protein) were normal. His liver and renal function was also normal. The lesion was surgically removed and the resected tissue was sent for histopathology, culture and polymerase chain reaction (PCR) testing for *Leishmania* parasites. Material from the lesion was macerated, under sterile conditions, and placed in Novy-MacNeal-Nicolle biphasic culture medium. The culture was negative for bacteria or parasites but PCR, using molecular screening methods: EF-PCR and k26-PCR, a molecular assay specific for the *L. donovani* complex that discriminates *L. donovani*/*L. infantum* zymodemes [7], revealed the presence of *L. donovani* in the tissue of the lesion of the patient. The histopathology report was unremarkable other than signs of chronic inflammation. Following surgical resection, a short course of intravenous liposomal amphotericin B was administered to the child at a dose of 3mg/kg/day for 7 days. The child had no side effects and no significant scar remained. The child was followed up for a period of 18 months with no relapse.

Case number 2, a 60-year-old Greek Cypriot woman (the child’s godmother who lives next door) developed a 2cm diameter erythematous papule on her glabella, simultaneously to this child’s illness. Culture and PCR of lesion material (as described above) were positive for *L. donovani*[7] and subsequent typing of the isolated parasites, using multilocus enzyme electrophoresis [8], provided the infraspecific position of the parasite as pertaining to MON-37 zymodeme [6,9]. She was treated at the Dermatology clinic of the University Hospital of Crete, Greece. She was given liposomal amphotericin B and local thermotherapy and completely recovered with a very faint scar at the site of the lesion. No recurrence was observed until today, 20 months later.

Case number 3, a 60-year-old Greek Cypriot man (the child’s godfather and husband of Case number 2) had developed a typical CL ulcer on his face, on the right, above the moustache area. He refused to visit the doctor and the lesion persisted for more than 1 year; it self-resolved leaving a small scar. No material was available for testing.

Case number 4, a 40-year-old Greek Cypriot woman (the child’s aunt) who resided in a house next to the child’s, had developed a lesion in her neck area approximately 1 year earlier. She had already been treated, with surgical removal of the lesion followed by liposomal amphotericin B and at the time of the study the scar was evident. The doctor who had treated her described the ulcer on her neck as a typical CL lesion.

## Discussion

Clustering of cases of CL or VL caused by anthroponotic species, such as *L. donovani*, especially within households, have been reported in large population studies in endemic countries [3,4,10]. We present a cluster of four cases of CL caused by *L. donovani* including a 6-year-old boy in Cyprus. Human cases of this anthroponotic species were detected for the first time in Europe in 2006 when the first six cases were diagnosed in the Republic of Cyprus [6]. To the best of our knowledge, this is the first case of CL by *L. donovani* in a child and the first cluster with adult cases of CL by *L. donovani* reported in Europe.

In Cyprus, there is a paradox with respect to *Leishmania* epidemiology since two different, parallel, transmission cycles appear to be involved. In the first transmission cycle, *L. infantum* causes canine leishmaniasis, with 11.9% overall seroprevalence in dogs. Although dog seropositivity for *L. infantum* reached 33.3% in some areas, no human VL or CL cases among Greek Cypriots have been detected [9]. The second transmission cycle is anthroponotic and all known cases of CL or VL are caused by *L. donovani*[6,11]. Many possible explanations have been proposed in order to explain the origin of *L. donovani* in Cyprus. Recent studies, by multilocus microsatellite typing, showed that the *L. donovani* MON-37, isolated from human cases in Cyprus, bear substantial genetic differences to *L. donovani* MON-37 isolates from India, Israel, Sri Lanka and Kenya [12]. However, they reveal genetic similarities to isolates from Turkey suggesting that the Cypriot isolates may have originated from that country [13].

Diagnosis of CL depends on the clinical signs, epidemiological information, PCR and culture of infected tissue, or finding the intracellular parasite in biopsy material from the periphery of the lesion. It is not always possible to culture the parasite (gold standard method) since other microorganisms from the wound may infect the culture material and stop *Leishmania* growth.

Treatment for CL depends on the *Leishmania* species involved [14] and the means available to the hospital. Not all patients require treatment since lesions may heal spontaneously, after approximately 1 year, leaving a skin scar [15]. *Leishmania* lesions appear to respond well to local treatment using cryotherapy, thermotherapy or surgical removal, in combination with chemical treatment; mainly meglumine antimoniate or amphotericin B but pentamidine isethionate, paromomycin, and antifungals are also used [15]. Antimonials have a high incidence of reversible adverse effects and since *Leishmania* drug resistance to meglumine antimoniate has been reported in some areas [16,17] it should be used with caution. Liposomal amphotericin B has proved effective in cases of leishmaniasis caused by a wide range of *Leishmania* species. In particular, it has proved effective for the treatment of paediatric VL caused by *L. infantum* in the Mediterranean region [18], and *L. donovani* in India [19].

The child, Case number 1, was managed successfully with surgical resection of the affected area and a short course of liposomal amphotericin B. Our decision to use the above treatment was supported by our previous experience of successfully treating a case of VL due to *L. donovani* MON-37 in an infant originating from a neighbouring village [6,9,11].

Treatment of CL is important because it limits skin scarring but it also reduces the risk of metastatic infection. For anthroponotic species, like *L. donovani* and *L. tropica*, treatment is very important in order to stop transmission from person to person through the reservoir.

## Conclusions

It appears that most of the CL and VL cases in Cyprus are found in a few villages in the district of Paphos. Detection of areas with clusters of cases may help in the study of the conditions favouring the epidemiology of the disease so that intervention measures could be targeted in high-risk areas. In our study, all four patients lived in three neighbouring houses, suggesting that transmission of the parasite occurred inside or in close proximity to these houses. The area in question is currently under investigation regarding the seasonal distribution of the sand fly fauna (the vector of the disease) in relation to the characteristics of the microenvironment which favour their survival, spread and contact with people. Lessons from this familial cluster and its environmental risk-factor assessment may prove beneficial to safeguard public health and implement successful preventive and public health measures that could also be applied in other areas and countries.

## Consent

Written informed consent was obtained from the patients for publication of this case report and any accompanying images. An additional written informed consent was obtained from the parents of Case number 1 for publication of this case report and the accompanying image. Copies of the written consents are available for review by the Editor-in-Chief of this journal.

## Competing interests

The authors declare that they have no competing interests.

## Authors’ contributions

MGK was responsible for the conception of the case report, acquisition of clinical data and clinical treatment. YA, VC, and AM contributed to the collection of epidemiological data regarding *Leishmania donovani*. MA performed the laboratory testing and interpreted the results. MGK and ESS drafted the case report. All authors contributed to the writing of the final manuscript. All authors read and approved the final manuscript.

## Author’s information

Dr Maria Koliou is a paediatric infectious disease specialist at the Archbishop Makarios Hospital in Cyprus. Her research interests include zoonoses affecting mainly the countries of the Mediterranean.
